# A two-stage cluster sampling method using gridded population data, a GIS, and Google Earth^TM^ imagery in a population-based mortality survey in Iraq

**DOI:** 10.1186/1476-072X-11-12

**Published:** 2012-04-27

**Authors:** LP Galway, Nathaniel Bell, Al Shatari SAE, Amy Hagopian, Gilbert Burnham, Abraham Flaxman, Wiliam M Weiss, Julie Rajaratnam, Tim K Takaro

**Affiliations:** 1 Faculty of Health Sciences, Simon Fraser University, Blusson Hall 8888 University Drive, Burnaby, B.C, Canada V5A 1 S6, USA; 2 Department of Surgery, University of British Columbia, West Mall, Vancouver, BC, Canada; 3 Human Development and Training Center, Iraqi, Ministry of Health, Iraq; 4 School of Public Health, University of Washington, Seattle, WA, USA; 5 School of Public Health, Johns Hopkins University, Baltimore, MD, USA

**Keywords:** Cluster sampling, Population-based survey, Mortality, Conflict, Iraq war, Geographic information system (GIS), Google Earth^TM^

## Abstract

**Background:**

Mortality estimates can measure and monitor the impacts of conflict on a population, guide humanitarian efforts, and help to better understand the public health impacts of conflict. Vital statistics registration and surveillance systems are rarely functional in conflict settings, posing a challenge of estimating mortality using retrospective population-based surveys.

**Results:**

We present a two-stage cluster sampling method for application in population-based mortality surveys. The sampling method utilizes gridded population data and a geographic information system (GIS) to select clusters in the first sampling stage and Google Earth ^TM^ imagery and sampling grids to select households in the second sampling stage. The sampling method is implemented in a household mortality study in Iraq in 2011. Factors affecting feasibility and methodological quality are described.

**Conclusion:**

Sampling is a challenge in retrospective population-based mortality studies and alternatives that improve on the conventional approaches are needed. The sampling strategy presented here was designed to generate a representative sample of the Iraqi population while reducing the potential for bias and considering the context specific challenges of the study setting. This sampling strategy, or variations on it, are adaptable and should be considered and tested in other conflict settings.

## Background

Monitoring civilian mortality in conflicts can help target humanitarian assistance and minimize loss of life among those caught up in conflict. While in stable situations surveillance of mortality using vital registration systems is the gold standard, these systems are rarely functional during conflicts and are often nonexistent where major conflicts occur [1]‐[3]. Alternative approaches, such as passive surveillance through news media or press reports have been shown to under-record deaths and may be distorted by political agendas [4]. Consequently, epidemiologists are often limited to estimating mortality using retrospective population-based surveys at the household level [5]. In these, a representative sample of consenting households is selected to assess mortality events over a given time period, and mortality rates, along with their upper and lower confidence intervals, are calculated [5]. These calculations can also be compared to other time points to estimate excess mortality related to a conflict. However, simple random and systematic random sampling methods are difficult during conflict given data unavailability and logistical and security constraints [6,7].

One alternative approach is to apply cluster sampling to estimate conflict-related mortality rates. Two-stage cluster sampling was standardized in 1978 by the World Health Organization's Expanded Programme on Immunization (WHO EPI) to assess vaccine coverage and has since been extended to estimate conflict-related mortality in Iraq [8,9], Kosovo [10], the Democratic Republic of Congo [11], and Sudan [12]. This approach is relatively fast, can be done with limited financial and human resources, and exposure to unsafe areas can be limited. Additionally, a complete sampling frame is not required. These are all very important considerations in conflict settings.

In conventional two-stage cluster sampling, the first sampling stage involves the selection of a predetermined number of clusters. Clusters are mutually exclusive subpopulations, most frequently constructed from recognized administrative boundaries [13]. Clusters are selected from a list of primary sampling units (e.g. census areas, township boundaries) with the probability of selection proportional to population size (or estimated population size) [14]. In the second stage, starting households are selected from each cluster. As complete and adequate listings of households rarely exist, households are not selected from a sampling frame. Rather, they are selected by the survey team in the field based on a random procedure [15]. Most commonly, starting households are selected in the field based on the “random walk” method, which involves identifying the center of the cluster, or another easily distinguishable feature such as a main street, and selecting a random direction to walk, thus drawing a transect across the cluster. In practice, the random direction is often selected by “spinning a pen” [16]. Among those households that lie along the transect, one household is randomly selected as the starting household and a predetermined number of next nearest households are surveyed. Ultimately, the data collected from each cluster are pooled to make inferences with respect to the target population and standard errors are adjusted for design effects of using a cluster sampling approach [6].

Despite the benefits of this WHO EPI-type cluster sampling, the validity of this approach has been questioned [17]. Most criticisms are related to the potential for bias in the second stage when using the “random walk” approach, which has been shown to introduce bias if the household selection procedure is not in fact random [7,14,16,18,19]. In addition, this approach is subject to interviewer bias, whether conscious or unconscious, and can take a significant amount of time to implement in the field. Too much field time exposes survey team members to risk in conflict settings. It is also impossible to calculate the probability of selection at the household level, so the sample is not a true probability sample [14].

To date, a few variations on this conventional cluster sampling approach have been developed for application in nation-wide health studies. Relevant examples that have been used and show promise for certain settings include compact segment sampling [14,20] and random spatial sampling using global positioning system (GPS) coordinates [16]. However, these approaches may not be appropriate in conflict settings. Compact segment sampling requires a significant amount of field time exposure and two visits to each cluster [15] while the use of GPS units is often a security risk in the context of modern warfare [9]. Variations on the conventional two-stage cluster sampling designed for nation-wide mortality estimates in conflict settings are needed to generate accurate and useful mortality estimates and to contribute to theoretical and practical advances in the field of conflict epidemiology [21].

This paper presents a two-stage cluster sampling method implemented in a retrospective mortality study in Iraq. Our goal was to develop a cluster-based sampling method while taking into consideration the specific challenges of conflict settings. Our cluster sampling uses a gridded population dataset and a spatial sampling algorithm in a geographic information system (GIS) to select clusters in the first stage. Starting households are selected in the second stage using imagery and a sampling grid in Google Earth ^TM^[22].

## Methods

We received University of Washington Institutional Review Board approval for the study, and also received approval from the Ministries of Health in Baghdad and in Kurdistan. Methods were reviewed to ensure they complied with the ethical guidelines for epidemiological research set out by the Council for International Organizations of Medical Sciences. An ethicist experienced in international research associated with the Institute of Translational Health Sciences at the University of Washington further reviewed the protocols to ensure the safety of subjects and interviewers was adequately protected. Additionally, Simon Fraser University's Research Ethics Board approved the use of secondary data for this project.

### Data and Tools

The sampling method uses a gridded population dataset in the first stage of sampling. As a preliminary step, we reviewed a number of spatial population datasets. To date, three high resolution global gridded population datasets have been generated and used in epidemiological studies: the Gridded Population of the World (GPWv3) [23], Global Rural–Urban Mapping Project (GRUMPv1) [23], and LandScan^TM^[24]. These datasets use different interpolation methods to generate gridded population counts (see Table 1). These differences are important in selecting the most appropriate spatial population dataset. Each dataset is publically available (for research and public health purposes), accessible online, and can be easily integrated into most GIS platforms. If available, an alternative is to use country-specific datasets, which exist for many countries in Africa (see Afripop Project, 2011 and United Nations Environment Programme Gridded Population Databases) [25,26]. We selected the LandScan^TM^ dataset for reasons described below.

**Table 1:** Overview of gridded population datasets currently available

**Dataset**	**Provider(website)**	**Spatial resolution**	**Input population data source**	**Interpolation method**	**Ancillary data**	**Year(s)**
GPWv3.0	CIESIN (http://sedac.ciesin.columbia.edu/gpw/)	2.5’(~5 km^2^)	UNPD census data	Areal weighting ^1^	-None	1990,1995, 2000,2005 (projection),2010 (projection), 2015 (projection)
GRUMPv1	CIESIN (http://sedac.ciesin.columbia.edu/gpw/)	.5’(~1 km^2^)	UNPD census data	Dasymetric mapping ^2^	-Night-time light imagery-Populated places	2000
LandScan^TM^	ORNL (http://www.ornl.gov/sci/landscan/)	.5’(~1 km^2^)	Population Division of the U.S. Census Bureau	Smart interpolation ^3^	-Land cover-Road networks-Digital elevation models-Slope-Satellite imagery	2008

Adapted from [27].

^1^ Areal weighting overlays a grid onto sub national administrative unit population data and distributes the population across space according to the proportion of the administrative unit area that is contained within the grid cell [28].

^2^ Dasymetric mapping disaggregates sub national population estimates into grid units using ancillary data such as road networks [28,29].

^3^ Smart interpolation disaggregates sub national population estimates to grid cells according to likelihood co-efficients of population occurrence derived from ancillary data such as proximity to roads, slope, land cover [30].

In our approach, the first cluster sampling stage uses the ‘Create Spatially Balanced Points’ (CSBP) function in the ArcGIS (v10) software platform. This tool uses a spatial sampling algorithm based on the work of Theobold et al. (2007) [31] and Stevens & Olsen (2004) [32]. It uses a probability surface depicting relative probabilities of inclusion and a Reversed Randomized Quadrant-Recursive Raster (RRQRR) algorithm to randomly generate a set of spatially balanced points [31,32]. The probabilities of inclusion can be based on any relevant attribute, but the use of population size enables the application of a probability proportional to estimated size (PPES) approach. When using population data to generate the probabilities of inclusion, a sample that mimics the distribution of the target population is generated. This allows researchers to analyze the final dataset without weighting or other constructs to create a full population estimate.

Administrative boundaries are required to implement the sampling strategy presented here. Current and spatially referenced administrative boundaries (at the country, provincial, and district scale) can be downloaded from the Global Administrative Areas website in shapefile format for nearly all nations in the world [33].

Imagery in the Google Earth ^TM^ platform is used in the second stage of the sampling method. Google Earth ^TM^ maps the earth by the superimposition of images obtained from satellite imagery and aerial photography (images from airplanes, kites and balloons) [34]. This is a particularly useful tool for public health and conflict epidemiology as it has no financial cost, is easy to use, and can interact with other mapping technologies. Although the resolution, quality, and age of the imagery varies across the globe, it is generally possible to identify individual household rooftops.

### Gridded Population Dataset Selection

We selected the 2008 LandScan^TM^ gridded population dataset to depict the population of Iraq. The 2008 LandScan^TM^ population data were obtained from the Oak Ridge National Laboratory [24]. This dataset was selected over others for these reasons:

· *Theory*: We preferred the “smart interpolation” approach over the areal weighting approach for the disaggregation of sub-national population counts to grid units. Smart interpolation uses numerous sources of ancillary data (i.e. land cover, road network, slope, etc.) and does not assume that populations are uniformly distributed across space within administrative units [35].

· *Timeliness*: The 2008 LandScan^TM^ dataset offered the most up to date spatial population dataset available. Both GRUMP and GPW were released in 2000. Since a census has not been conducted in Iraq for decades, all population datasets are based on out-dated census information. However, the ancillary data used to disaggregate population data, land cover data for example, is most recent for the LandScan^TM^ dataset.

· *Validation in study area*: Mubareka’s 2008 study conducted in Northern Iraq found the LandScan^TM^ dataset correlated with settlements and population distribution on the ground [36].

· *Validation in other conflict settings and limited resource areas*: A review entitled “Tools and Methods for Estimating Populations at Risk from Natural Disasters and Complex Humanitarian Crises” recommended that the LandScan^TM^ population estimates tend to be better than other population sources in countries where the census data are spatially coarse and not recent, which is the case in Iraq [37].

### Stage 1- Cluster Selection

**Figure 1:**
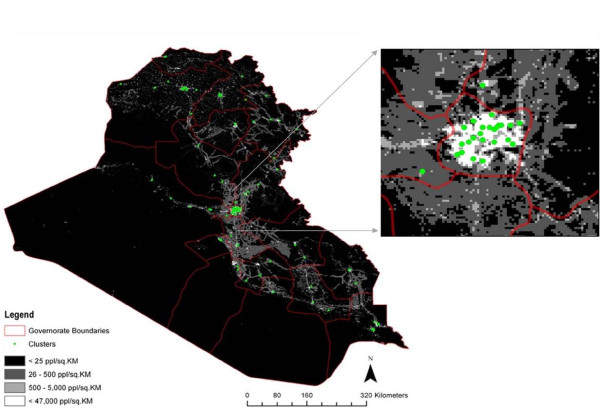
** Map of Iraq illustrating clusters in first sampling stage with governorate borders in red.** Inset is Baghdad area.

In the first stage of sampling, we used the 2008 LandScan^TM^ gridded population dataset and the CSBP tool to randomly select a sample of clusters weighted by estimated population size. The 2008 LandScan^TM^ gridded population dataset was downloaded in ESRI Grid format at the global scale, masked to the spatial extent of the Iraq administrative boundary and converted to a density grid. Using this raster (see Figure 1), we identified those grid cells with 25 people per km^2^ or fewer. These grid cells were assigned a probability of zero as they were unlikely to contain the minimum 20 households required for the survey design. For all other grid cells, we standardized the population dataset to create a probability surface. The probability surface is a raster layer with values ranging from 0 to 1, indicating the probability of inclusion for each grid cell. Higher values indicate a higher probability of inclusion. Here the probability of inclusion is based on population estimates according to LandScan^TM^ 2008 data.

We then used the CSBP tool to randomly generate 125 points according to the probability surface and the RRQRR algorithm. The grid cells containing one of the 125 points were then selected as possible clusters and numbered 1–125. Next, the 125 selected clusters were exported as a KML (Keyhole Markup Language) file for use in the Google Earth ^TM^ platform. Using the Google Earth ^TM^ satellite imagery, we visually examined all 125 clusters to identify any that were clearly not residential areas. Among these original 125 clusters, 12 were obviously either industrial areas, commercial areas, or otherwise not residential and were thus excluded from the initial sample set. Using the remaining 113 suitable clusters, we numbered the first 100 and held the remaining 13 clusters as a set of “backup” clusters that could replace any cluster deemed unsafe or otherwise inaccessible at the time of the survey.

### Stage 2- Starting Household Selection

**Figure 2:**
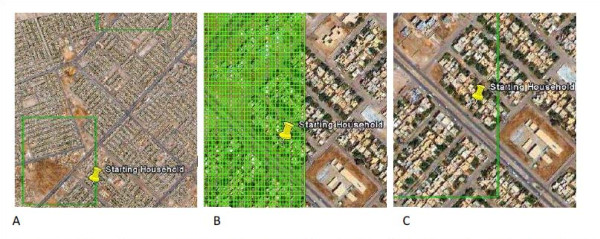
** Illustrative example of Starting Household selection and map used to locate Starting Household. A.** Illustrative cluster; **B.** Illustrative cluster with sampling grid; **C.** Illustrative starting household. The cluster and selected household shown here are for illustrative purposes only to protect the identity of households that participated in the survey. The image of the sampling grid shows only a small proportion of the entire grid. The imagery is from Google Earth^TM^.

 In the second stage of sampling, we needed to select a starting household in each of the first-stage clusters. The starting households were randomly selected using a sampling grid superimposed over satellite imagery in the Google Earth ^TM^ platform (see Figure 2). Sampling grids were generated in ArcGIS at a resolution of 10 meters by 10 meters (10 m by 10 m) that corresponded to the spatial extent of each cluster. A resolution of 10 m by 10 m was selected for the sampling grid to approximate the extent of a single rooftop. These sampling grids were exported as KML files and subsequently superimposed onto satellite imagery in Google Earth^TM^. We assigned each 10 m by 10 m cell within the sampling grid a unique number, enabling the selection of a single grid cell using a random number generator. If that grid cell contained a household, ascertained through visual assessment of satellite imagery, that household was marked using the “Add Placemark” tool. If the grid cell did not contain a household, we moved to the next randomly selected grid cells until a cell containing a household was selected. In the event that more than a single rooftop existed within the selected grid cell, the household with a greater proportion of its rooftop contained within the cell was selected. The same steps were followed to select “back-up” starting households in each cluster in the event that a household no longer existed, was not a residential building, or was not accessible for security reasons.

## Results and Discussion

### Application: Iraq 2011 University Collaborative Mortality Study

This sampling method was designed for a retrospective population-based study aimed at estimating mortality in Iraq: the Iraq 2011 University Collaborative Mortality. This study was carried out in 2011 and was designed to update and extend earlier mortality estimates published in 2004 [9], 2006 [8], and 2008 [38]. The 2011 Iraq mortality study used a standard household demographic method and a sibling survival technique [15,39]. Results of the Iraq 2011 University Collaborative Mortality Study are expected in 2012. A power calculation informed the original sampling design, leading to the selection of 100 clusters with 20 households per cluster, for a plan to sample 2,000 households.

### Survey Preparation and Implementation

GPS devices could not be used to locate starting households as laws prohibiting the use of this technology by Iraqi civilians made this an unacceptable security risk. Consequently, printed maps were used. For each cluster, we created maps at different scales. To help survey teams locate households efforts were made to produce maps that included easily identifiable structures such as water towers, highways, or mosques. As part of five day initial field training, two days were spent in map reading and orienteering. Teams quickly became adept at locating clusters and households using maps, which was verified in a second training session.

The 2011 Iraq mortality survey was carried out between May and July 2011 by four teams of Iraqi medical doctors with training in family medicine and community medicine and previous household survey experience. There were two supervisors each managing and monitoring the work and progress of two of the survey teams. We were fortunate that one of our project supervisors was particularly enthusiastic and adept in the use of maps. We also employed a person in each governorate who had local knowledge of the landscape. Only one (0.01%) of the 100 clusters selected for the original sample could not be visited because of safety concerns; this cluster was replaced with one of the remaining 13 “back-up” clusters from the same governorate.

### Feasibility and Methodological quality

In designing a cluster survey trade-offs and contextual factors must be considered to balance feasibility and methodological quality [40]. As there are few alternatives to cluster samples in conflict situations our goal was to improve methodological quality in a practical and feasible field approach. We believe that this adapted two-stage cluster sampling method meets these goals.

#### Feasibility

The feasibility of doing research in conflict-settings is always balanced by the requirement for rigorous protocols [41]. Factors influencing feasibility include survey team safety, time required for research, resources and expertise needed, and financial costs.

*Safety of survey team: S*afety of the survey team is not merely related to feasibility but is also an important ethical consideration [41]. In our study, the use of GPS to identify starting households was viewed as a significant safety threat; this is likely to be the case in other conflict settings. The maps allowed teams to move directly to the starting household without the extended exposure needed for the “random walk” method. Using a local facilitator to help locate starting households can reduce risk to the survey team.

*Time*: The element of time must be considered in terms of both preparation time (generating a sample selection prior to field work) and the amount of time in the field. Selection of the clusters, which included gathering and preparing the data used, took approximately 1 week for a single researcher. The process of selecting households and preparing the maps required approximately 2 weeks for a single researcher. Time needed in the field tended to vary depending on the setting. If the starting household was located in an urban setting or near an identifiable landmark, the household was identified quickly and with ease. This task was more time consuming and difficult for remote settings. The use of a local person with knowledge of the territory facilitated the task of locating a starting household. The ability to examine cluster and household locations before going into the field using Google Earth^TM^ proved helpful to field teams, and in areas where access was restricted by checkpoints and barriers permitted scouts to locate the site a day in advance and guide the survey teams the following day.

*Resources and expertise*: All of the datasets used are free and publically available for research purposes. The same is true for the Google Earth^TM^. ArcGIS however, requires a purchased licence. While expertise in GIS software is needed to carry out the steps in the two first stages of sampling, comparably less training is required to orientate survey teams in map reading and orienteering. We recommend employing at least one ground-level supervisor with a knack for geography and maps.

*Financial cost:* The financial costs related to the implementation of this sampling method are not large. Although the costs associated with the GIS software can be substantial, most institutions have some access to GIS software. Additionally, free or low-cost alternatives to ArcGIS could be considered (e.g. GRASS) [42,43]. Free and high-quality imagery from Google Earth^TM^ produces substantial savings over commercial imagery providers. Additionally, using maps rather than purchasing GPS units is a cost savings approach. Even in situations where GPS units are not a security concern the use of printed maps is a low-cost alternative for localizing sites [44].

#### Methodological quality

Tapp et al. [2008] present quality indicators for retrospective mortality surveys in complex emergencies [45]. Two of the five indicators, coverage and bias, are specifically related to sampling design and are discussed below in relation to the method presented here.

*Coverage*: Is the sample sufficiently representative of the underlying population of interest [45]? To answer this question, we examined the coverage of our sample in terms of regional distribution across administrative units and urban–rural status in comparison to the Iraqi population in general (see Table 2). Regional distribution and urban–rural status are important factors, as violence and therefore mortality risk are presumably influenced by both. We examined the percentage of total clusters and households in each governorate (an administrative unit akin to a province or state) compared to the percentage of the Iraqi population in each governorate. These comparisons suggest that our sample sufficiently captures the regional distribution of the underlying Iraqi population. The extent of urban–rural coverage of our sample was assessed by identifying those clusters located in urban regions and those located in rural regions and comparing the urban–rural proportion of our clusters to the national urban–rural proportion. We classified clusters as urban or rural based on local knowledge of survey team supervisors as there were no adequate data available identifying urban–rural status at the local level. Urban regions are defined here as metropolitan cities, towns, or peri-urban areas while rural areas are defined as remote communities with low population density. Based on best available estimates, the urban–rural divide in Iraq is approximately 66 percent urban to 34 percent rural (UN population data [46], US census Bureau [47], and Iraq’s Central Organization for Statistics and Information [48] all provide estimates between 66 percent and 67 percent) According to our classification of clusters as urban or rural, 31 percent of the sample is rural while 69 percent is urban. Our sample has slightly over-sampled the urban population, which is to be expected as we excluded the very remote areas in our sampling strategy to ensure that cluster locations had at least 20 households. Nonetheless, the urban–rural coverage in our study is very close to the estimated national average.

**Table 2:** Sample selection clusters across Iraqi governorates for 2011 Iraq mortality study, compared to Iraq population estimates

**Governorates**	**Estimated population^1^**	**Percentage of Iraqi population**	**No. of clusters**	**Percentage of sample**	**Difference (pop% - sample%)**	**No. of individuals^2^**	**Percentage of sample**	**Difference (pop% - sample%)**
Al-Anbar	1,451,583	4.52	7	7	−2.48	990	9.28	−4.76
Al-Muthanna	719,824	2.24	1	1	1.24	142	1.33	0.91
Al-Najaf	1,180,681	3.68	2	2	1.68	200	1.88	1.80
Al-Qadisiya	1,121,782	3.49	4	4	−0.51	580	5.44	−1.95
Babil	1,727,032	5.38	3	3	2.38	353	3.31	2.07
Baghdad	7,180,889	22.37	23	23	−0.63	2,347	22.01	0.36
Basrah	2,555,542	7.96	8	8	−0.04	882	8.27	−0.31
Diala	1,370,537	4.27	5	5	−0.73	463	4.34	−0.07
Duhouk	968,901	3.02	2	2	1.02	284	2.66	0.36
Erbil	1,471,053	4.58	9	9	−4.42	803	7.53	−2.95
Kerbala	1,003,516	3.13	2	2	1.13	219	2.05	1.08
Kirkuk	1,290,072	4.02	2	2	2.02	201	1.89	2.13
Missan	1,009,565	3.14	3	3	0.14	308	2.89	0.25
Ninevah	3,237,918	10.09	13	13	−2.91	1,298	12.17	−2.08
Salahuddin	1,259,298	3.92	3	3	0.92	312	2.93	0.99
Sulaimaniya	1,551,974	4.83	7	7	−2.17	663	6.22	−1.39
Thi Qar	1,846,788	5.75	3	3	2.75	308	2.89	2.86
Wasit	1,158,033	3.61	3	3	0.61	310	2.91	0.70

^1^ Estimated 2009 population According to Central organization for statistics and Information Technology [COSIT] estimates.

^2^ Total number of individuals based on reported household members.

*Bias*: As Tapp et al. (2008) highlight [45] an important quality consideration for retrospective surveys is whether the population was sampled to avoid bias.

The sampling method presented here was designed to reduce the potential for sampling bias by randomly-selecting both clusters and households prior to field work. We also took into account criticisms regarding the sampling strategy used in the 2006 Iraq mortality study, especially what some authors have referred to as a “main street bias.” Using sampling grids enables simple random selection of households to minimize the possibility of systematic selection of certain regions and further removes the possibility of conscious or unconscious interviewer bias by selecting households a priori.

Nevertheless, the sampling method described here does have several potential sources of bias, mostly stemming from the population size from which clusters were defined [17]. The LandScan 2008^TM^ population data for Iraq is not based on official 2008 census data, but rather uses estimated population figures derived from the most recent national census. A complete national census has not been conducted in Iraq since 1987 (although a partial census excluding Kurdish regions was carried out in 1997). A full Iraqi census has been planned since the early 2000’s but has been repeatedly postponed [48]. Both internal and external displacement have been significant in Iraq since the US-led invasion in 2003; it is unlikely that any extrapolated population estimate has accurately captured the complex patterns of population movement [49]. It is estimated that there are up to 2.5 million Iraqis who have fled Iraq to neighbouring countries and at least that number who have internally migrated principally due to violence [50]. It is difficult to estimate how the use of out-dated population figures and migration affects sampling and the potential for bias. It is likely that there are important implications, perhaps with mortality in the most violent regions underestimated, and violence in areas receiving internally displaced being overstated.

The construction method of the LandScan^TM^ dataset may also introduce bias. The smart interpolation approach employed by LandScan^TM^ uses ancillary data such as road networks and satellite imagery to define occupation probabilities for all grid cells in a raster grid [51]. Assumptions made in generating these grid cell occupation probabilities, that people are most likely to live along roads for example, may not reflect true population distribution on the ground in some areas. In the field we did not encounter problems due to this theoretical limitation.

Lastly, the use of Google Earth^TM^ imagery may also introduce bias as images can be out-dated, thereby excluding recent development. This is especially problematic if the newly developed communities have differential mortality experiences. In our study, imagery dates range from 2002 to 2011, with the majority (70%) from late 2004. It is possible that households not captured in older imagery could be families fleeing more violent areas and therefore families with higher probability of mortality events. Google Earth^TM^ satellite imagery may thereby select households that underestimate mortality. It is also important to note that Google Earth^TM^ satellite imagery does not cover every region of the globe with high resolution images such that individual rooftops may not be identifiable. If this is the case, alternative sources of imagery may be needed.

We should note the measures of bias are both objective and subjective. Our own surveyors expressed concerns that this study sample appeared to minimize the enumeration of mortality events by the choice of too many clusters in remote areas and potentially protected populations (such as oil worker company enclaves) and not capturing mortality events among families that fled the country.

## Conclusion

For a variety of political and economic failures, conflict affects many populations around the world. Given increasing resource constraints, economic instability, dwindling oil supplies, and food and water stress due to climate change, global conflicts are not likely to decrease. There is a dearth of published work regarding both the population impacts of conflict and appropriate methods for studying the public health effects of conflict [41]. Although subject to certain limitations, retrospective population-based mortality studies are an important tool in conflict epidemiology. Sampling is a challenge in such studies and alternatives that improve on the conventional cluster approach are needed. As Morris & Nguyen note in their review of cluster sampling used in humanitarian emergencies, we need to “look beyond the standard methods for measuring mortality” [6]. Adapting conventional cluster sampling and using novel data sources, tools, and technologies can improve the overall validity of retrospective survey estimates and support the feasibility of research in challenging conflict settings. The sampling strategy presented here was designed to generate a sample representative of the Iraqi population. We sought to reduce the potential for bias while considering the context specific challenges of the study setting. When designing sampling methods for retrospective population-based mortality surveys, researchers must consider all available methods, options for improving and adapting these methods for a particular setting and endpoint, and the implications for feasibility and study validity. This sampling strategy, or variations on it, are adaptable and should be tested in other conflict settings.

## Competing interests

The authors declare that they have no competing interests with regards to this manuscript.

## Authors’ contributions

All authors participated in the design and conceptualization of the sampling method. LPG carried out the sampling steps and wrote the initial draft of the manuscript. All authors provided valuable feedback on the method and contributed to the writing of the manuscript. All authors read and approved the final manuscript.

## References

[B1] Obermeyer Z Murray CJL Gakidou E Fifty years of violent war deaths from Vietnam to Bosnia: analysis of data from the world health survey programme BMJ 2008 336 1482 1486 10.1136/bmj.a137 18566045 PMC2440905

[B2] Levy BS Sidel VW War and public health 1997 USA: Oxford University Press

[B3] State violence in Guatemala, 1960–1996: a quantitative reflection http://shr.aaas.org/guatemala/ciidh/qr/spanish/contents.html

[B4] Roberts L Commentary: Ensuring health statistics in conflict are evidence-based Confl Health 2010 4 10 10 10.1186/1752-1505-4-10 20444287 PMC2874520

[B5] Mills EJ Checchi F Orbinski JJ Schull MJ Burkle FM Beyrer C Cooper C Hardy C Singh S Garfield R others: Users’ guides to the medical literature: how to use an article about mortality in a humanitarian emergency Conflict and Health 2008 2 9 10.1186/1752-1505-2-9 18826636 PMC2569008

[B6] Morris SK Nguyen CK A review of the cluster survey sampling method in humanitarian emergencies Public Health Nurs 2008 25 370 374 10.1111/j.1525-1446.2008.00719.x 18666943

[B7] Checchi F Roberts L Documenting Mortality in Crises: What Keeps Us from Doing Better Plos Med 2008 5 e146 10.1371/journal.pmed.0050146 18597552 PMC2443202

[B8] Burnham G Lafta R Doocy S Roberts L Mortality after the 2003 invasion of Iraq: a cross-sectional cluster sample survey The Lancet 2006 368 1421 1428 10.1016/S0140-6736(06)69491-9 17055943

[B9] Roberts L Lafta R Garfield R Khudhairi J Burnham G Mortality before and after the 2003 invasion of Iraq: cluster sample survey The Lancet 2004 364 1857 1864 10.1016/S0140-6736(04)17441-2 15555665

[B10] Spiegel PB Salama P War and mortality in Kosovo, 1998–99: an epidemiological testimony The Lancet 2000 355 2204 2209 10.1016/S0140-6736(00)02404-1 10881894

[B11] Coghlan B Brennan RJ Ngoy P Dofara D Otto B Clements M Stewart T Mortality in the Democratic Republic of Congo: a nationwide survey The Lancet 2006 367 44 51 10.1016/S0140-6736(06)67923-3 16399152

[B12] Depoortere E Checchi F Broillet F Gerstl S Minetti A Gayraud O Briet V Pahl J Defourny I Tatay M Brown V Violence and mortality in West Darfur, Sudan (2003–04): epidemiological evidence from four surveys The Lancet 2004 364 1315 1320 10.1016/S0140-6736(04)17187-0 15474133

[B13] Hoshaw-Woodard S Description and comparison of the methods of cluster sampling and lot quality assurance sampling to assess immunization coverage 2001 Organization: World Health

[B14] Turner AG Magnani RJ Shuaib MA Not Quite as Quick but Much Cleaner Alternative to the Expanded Programme on Immunization (EPI) Cluster Survey Design International Journal of Epidemiology 1996 25 198 203 10.1093/ije/25.1.198 8666490

[B15] Rose A Grais R Coulombier D Ritter H A comparison of cluster and systematic sampling methods for measuring crude mortality Bull. World Health Organ 2006 84 290 296 16628302 10.2471/blt.05.029181 PMC2627322

[B16] Grais F Rose A Guthmann J Don’t spin the pen: two alternative methods for second-stage sampling in urban cluster surveys 2007 10.1186/1742-7622-4-8 PMC1894792 17543102

[B17] Working group for Mortality Estimation in Emergencies Wanted: studies on mortality estimation methods for humanitarian emergencies, suggestions for future research Emerg Themes Epidemiol 2007 4 9 17543103 10.1186/1742-7622-4-9 PMC1904216

[B18] Guha-Sapir D Degomme O Estimating mortality in civil conflicts: lessons from Iraq 2007 Brussles: Centre for Research on the Epidemiology of Disasters

[B19] Spiegel PB Robinson C Large-Scale“Expert” Mortality Surveys in Conflicts–Concerns and Recommendations JAMA 2010 304 567 10.1001/jama.2010.1094 20682938

[B20] Luman ET Worku A Berhane Y Martin R Cairns L Comparison of two survey methodologies to assess vaccination coverage International Journal of Epidemiology 2007 36 633 641 10.1093/ije/dym025 17420165

[B21] Bostoen K Bilukha O Fenn B Morgan O Tam C ter Veen A Checchi F Methods for health surveys in difficult settings: charting progress, moving forward Emerging Themes in Epidemiology 2007 4 13 10.1186/1742-7622-4-13

[B22] Google Earth http://www.google.com/earth/index.html#utm_campaign=en&utm_medium=ha&utm_source=en-ha-na-us-bk-eargen&utm_term=googel%20earth

[B23] Gridded Population of the World - GPW v3 http://sedac.ciesin.columbia.edu/gpw/

[B24] LandScan Home http://www.ornl.gov/sci/landscan/

[B25] Linard C Gilbert M Snow RW Noor AM Tatem AJ Population Distribution, Settlement Patterns and Accessibility across Africa in 2010 PLoS ONE 2012 7 e31743 10.1371/journal.pone.0031743 22363717 PMC3283664

[B26] Tatem AJ Noor AM von Hagen C Di Gregorio A Hay SI High Resolution Population Maps for Low Income Nations: Combining Land Cover and Census in East Africa PLoS ONE 2007 2 e1298 10.1371/journal.pone.0001298 18074022 PMC2110897

[B27] Tatem AJ Campiz N Gething PW Snow RW Linard C The effects of spatial population dataset choice on estimates of population at risk of disease Popul Health Metrics 2011 9 4 10.1186/1478-7954-9-4 PMC3045911 21299885

[B28] Mennis J Generating surface models of population using dasymetric mapping The Professional Geographer 2003 55 31 42

[B29] Balk DL Deichmann U Yetman G Pozzi F Hay SI Nelson A Determining Global Population Distribution: Methods, Applications and Data. In Global Mapping of Infectious Diseases: Methods, Examples and Emerging Applications Academic Press 2006 62 119 156 10.1016/S0065-308X(05)62004-0 PMC3154651 16647969

[B30] Dobson JE Bright EA Coleman PR Durfee RC Worley BA LandScan: a global population database for estimating populations at risk Photogrammetric Engineering and Remote Sensing 2000 66 849 857

[B31] Theobald DM Stevens DL White D Urquhart NS Olsen AR Norman JB Using GIS to Generate Spatially Balanced Random Survey Designs for Natural Resource Applications Environmental Management 2007 40 134 146 10.1007/s00267-005-0199-x 17546523

[B32] Stevens DL Olsen AR Spatially balanced sampling of natural resources Journal of the American Statistical Association 2004 99 262 278 10.1198/016214504000000250

[B33] Global Administrative Areas http://www.gadm.org/country

[B34] Kamadjeu R Tracking the polio virus down the Congo River: a case study on the use of Google EarthTM in public health planning and mapping International Journal of Health Geographics 2009 8 4 10.1186/1476-072X-8-4 19161606 PMC2645371

[B35] Salvatore M Pozzi F Huddleston B Bloise M Mapping global urban and rural population distributions 2005 Rome: FAO

[B36] Mubareka S Ehrlich D Bonn F Kayitakire F Settlement location and population density estimation in rugged terrain using information derived from Landsat ETM and SRTM data International Journal of Remote Sensing 2008 29 2339 2357 10.1080/01431160701422247

[B37] Tools and Methods for Estimating Populations at Risk from Natural Disasters and Complex Humanitarian Crises 2007 Washington, D.C: National Academies Press

[B38] Violence-Related Mortality in Iraq from 2002 to 2006 New England Journal of Medicine 2008 358 484 493 18184950 10.1056/NEJMsa0707782

[B39] Gakidou E King G Death by survey: estimating adult mortality without selection bias from sibling survival data Demography 2006 43 569 585 10.1353/dem.2006.0024 17051828

[B40] Edward F Peter B Population survey sampling methods in a rural African setting: Measuring mortality Population Health Metrics 2006 6 10.1186/1478-7954-6-2 PMC2440730 18492246

[B41] Ford N Mills EJ Zachariah R Upshur R Ethics of conducting research in conflict settings Confl Health 2009 3 7 7 10.1186/1752-1505-3-7 19591691 PMC2717053

[B42] GRASS GIS - The World Leading Free Software GIS http://grass.fbk.eu/

[B43] Boulos M Web GIS in practice III: creating a simple interactive map of England’s strategic Health Authorities using Google Maps API, Google Earth KML, and MSN Virtual Earth Map Control International Journal of Health Geographics 2005 4 22 10.1186/1476-072X-4-22 16176577 PMC1242244

[B44] Chang A Parrales M Jimenez J Sobieszczyk M Hammer S Copenhaver D Kulkarni R Combining Google Earth and GIS mapping technologies in a dengue surveillance system for developing countries International Journal of Health Geographics 2009 8 49 10.1186/1476-072X-8-49 19627614 PMC2729741

[B45] Tapp C Burkle FM Wilson K Takaro T Guyatt GH Amad H Mills EJ Iraq War mortality estimates: A systematic review Confl Health 2008 2 1 1 10.1186/1752-1505-2-1 18328100 PMC2322964

[B46] United Nations: Country profile, Iraq http://data.un.org/CountryProfile.aspx?crName=Iraq

[B47] CIA The World Factbookhttps://www.cia.gov/library/publications/the-world-factbook/geos/iz.html

[B48] Central Organization for Statistics and Information Technology http://cosit.gov.iq/english/

[B49] Lischer SK Security and Displacement in Iraq: Responding to the Forced Migration Crisis International Security 2008 33 95 119 10.1162/isec.2008.33.2.95

[B50] Margesson R Iraqi Refugees and Internally Displaced Persons: A Deepening Humanitarian Crisis 2008

[B51] Hay SI Noor AM Nelson A Tatem AJ The accuracy of human population maps for public health application Tropical Medicine & International Health 2005 10 1073 1086 10.1111/j.1365-3156.2005.01487.x 16185243 PMC3173846

